# Retraction Note: *Astragali radix*: could it be an adjuvant for oxaliplatin-induced neuropathy?

**DOI:** 10.1038/s41598-023-37011-5

**Published:** 2023-06-20

**Authors:** Lorenzo Di Cesare Mannelli, Alessandra Pacini, Laura Micheli, Angelo Pietro Femia, Mario Maresca, Matteo Zanardelli, Alfredo Vannacci, Eugenia Gallo, Anna Rita Bilia, Giovanna Caderni, Fabio Firenzuoli, Alessandro Mugelli, Carla Ghelardini

**Affiliations:** 1 Department of Neuroscience, Psychology, Drug Research and Children’s Health - NEUROFARBA - Pharmacology and Toxicology Section, Florence, Italy; 2 Department of Experimental and Clinical Medicine - DECM - Section of Anatomy and Histology, University of Florence, Florence, Italy; 3 Department of Chemistry, University of Florence, Florence, Italy

Retraction of: *Scientific Reports* 10.1038/srep42021, published online 10 February 2017

The Editors have retracted this Article.

After publication of this Article it was brought to the Editors’ attention that some of the data appear to overlap with data in other articles from the same author group, where the data is partially attributed to different samples. Specifically:

- In Figure 3B the “vehicle + 50% HA” group appears to overlap with data in Figure 8 published in^1^;
- In Figure 3B the “oxaliplatin + 50% HA” group appears to overlap with data in Figure 4a published in^2^;
- In Figure 3C the “vehicle + 50% HA” group appears to overlap with data in Figure 4 published in^3^;
- In Figure 4A the “vehicle + 50% HA” group appears to overlap with data in Figure 7d “vehicle + vehicle cg1” group published in^2^;
- In Figure 4A the “vehicle + vehicle” group appears to overlap with data in Figure 7d “vehicle + vehicle M1” group published in^2^.

The Editors reached out to the Authors to request raw data. The Authors were unable to provide the data and the concerns remain unresolved. The Editors therefore no longer have confidence in the results presented in this Article.

All Authors agree with the retraction of the Article.

## References

[1] Di Cesare ML, Pacini A, Micheli L, Zanardelli M, Ghelardini L (2014). Glial role in oxaliplatin-induced neuropathic pain. Exp. Neurol..

[2] Pacini A, Micheli L, Maresca M, Branca JJV, McIntosh JM, Ghelardini C, Di Cesare Mannelli L (2016). The α9α10 nicotinic receptor antagonist α-conotoxin RgIA prevents neuropathic pain induced by oxaliplatin treatment. Exp. Neurol..

[3] Di Cesare ML, Pacini A, Bonaccini L, Zanardelli M, Mello T, Ghelardini C (2012). Morphologic features and glial activation in rat oxaliplatin-dependent neuropathic pain. TJ. Pain.

